# DESTINATION: a phase 3, multicentre, randomized, double-blind, placebo-controlled, parallel-group trial to evaluate the long-term safety and tolerability of tezepelumab in adults and adolescents with severe, uncontrolled asthma

**DOI:** 10.1186/s12931-020-01541-7

**Published:** 2020-10-21

**Authors:** Andrew Menzies-Gow, Sandhia Ponnarambil, John Downie, Karin Bowen, Åsa Hellqvist, Gene Colice

**Affiliations:** 1 Royal Brompton Hospital, Sydney Street, London, SW3 6NP UK; 2 Late Respiratory and Immunology, BioPharmaceuticals R&D, AstraZeneca, Cambridge, UK; 3 Amgen, Thousand Oaks, CA USA; 4 Biometrics, Late Respiratory and Immunology, BioPharmaceuticals R&D, AstraZeneca, Gaithersburg, MD USA; 5 Biometrics, Late Respiratory and Immunology, BioPharmaceuticals R&D, AstraZeneca, Gothenburg, Sweden; 6 Late Respiratory and Immunology, BioPharmaceuticals R&D, AstraZeneca, Gaithersburg, MD USA

**Keywords:** Clinical trial, Long-term extension, Severe asthma, Tezepelumab

## Abstract

**Background:**

Tezepelumab is a human monoclonal antibody that blocks the activity of the epithelial cytokine thymic stromal lymphopoietin. The efficacy, safety and oral corticosteroid-sparing potential of tezepelumab are being investigated in two ongoing, phase 3, randomized, double-blind, placebo-controlled studies (NAVIGATOR [NCT03347279] and SOURCE [NCT03406078]). DESTINATION (NCT03706079) is a long-term extension (LTE) of these studies.

**Methods:**

DESTINATION is a randomized, double-blind, placebo-controlled LTE study in adults (18–80 years old) and adolescents (12–17 years old) with severe, uncontrolled asthma who are receiving treatment with medium- or high-dose inhaled corticosteroids plus at least one additional controller medication with or without oral corticosteroids. The study population will comprise patients who complete the 52- and 48-week NAVIGATOR and SOURCE studies, respectively. Patients who were randomized to receive tezepelumab 210 mg every 4 weeks (Q4W) in either predecessor study will continue to receive this regimen for 1 year; those who were previously randomized to receive placebo will be re-randomized (1:1) to receive either tezepelumab 210 mg Q4W or placebo for 1 year. Patients will receive their prescribed controller medications throughout DESTINATION and study physicians will have the opportunity to down- or up-titrate dosage of these medications, if appropriate. The primary objective is to evaluate the long-term safety and tolerability of tezepelumab over 104 weeks (inclusive of the treatment period of either predecessor study). The secondary objective is to assess the long-term effect of tezepelumab on asthma exacerbations. Patients recruited from SOURCE will be followed up post-treatment for 12 weeks. Patients recruited from NAVIGATOR who complete 100 weeks of tezepelumab treatment will be eligible for either 12 weeks of follow-up or a 36-week extended follow-up during which the clinical benefit of tezepelumab after treatment cessation will be investigated.

**Discussion:**

DESTINATION will evaluate the long-term safety, tolerability and efficacy of tezepelumab versus placebo with continued dosing for up to 2 years. DESTINATION will also evaluate the clinical effect of tezepelumab after treatment cessation. This LTE study aims to elucidate the long-term safety implications of receiving tezepelumab and to assess its potential long-term treatment benefits in patients with severe, uncontrolled asthma.

**Trial registration:**

NCT03706079 (ClinicalTrials.gov). Registered 15 October 2018.

## Background

Biological therapies targeting type 2 (T2) inflammatory cytokines, comprising anti-immunoglobulin (Ig)E and anti-interleukin (IL)-4/IL-13, IL-5 and IL-5R, have emerged as promising treatment options for patients with severe asthma [1]; however, data on the long-term safety and efficacy of these treatments are primarily derived from open-label, single-arm clinical trials [2–7]. The majority of these trials are limited by the lack of inclusion of a placebo group with which to compare the effects of biologic treatment and to support interpretation of the findings. The inclusion of a control group would also have provided further contextualization of pharmacologically unexpected adverse events [8]. Although double-blind extension studies of biologics for the treatment of severe asthma have been conducted, these have been limited to extended treatment periods of less than 1 year [9, 10]. Data on the effect of treatment cessation beyond the typical 12-week safety follow-up period in clinical trials are also generally lacking for available biologics.

Thymic stromal lymphopoietin (TSLP) is an epithelial-derived cytokine produced in response to environmental and inflammatory stimuli (including allergens, viruses and other airborne particles) that is implicated in the initiation and maintenance of airway inflammation [11–13]. TSLP acts upstream of T2 inflammatory cytokines and regulates multiple downstream inflammatory cascades [11]. It also has effects on airway structural cells [14–18] and may contribute to neutrophilic inflammation [19, 20]. The expression of TSLP is elevated in the airways of patients with asthma and correlates with disease severity [21, 22].

**Fig. 1:**
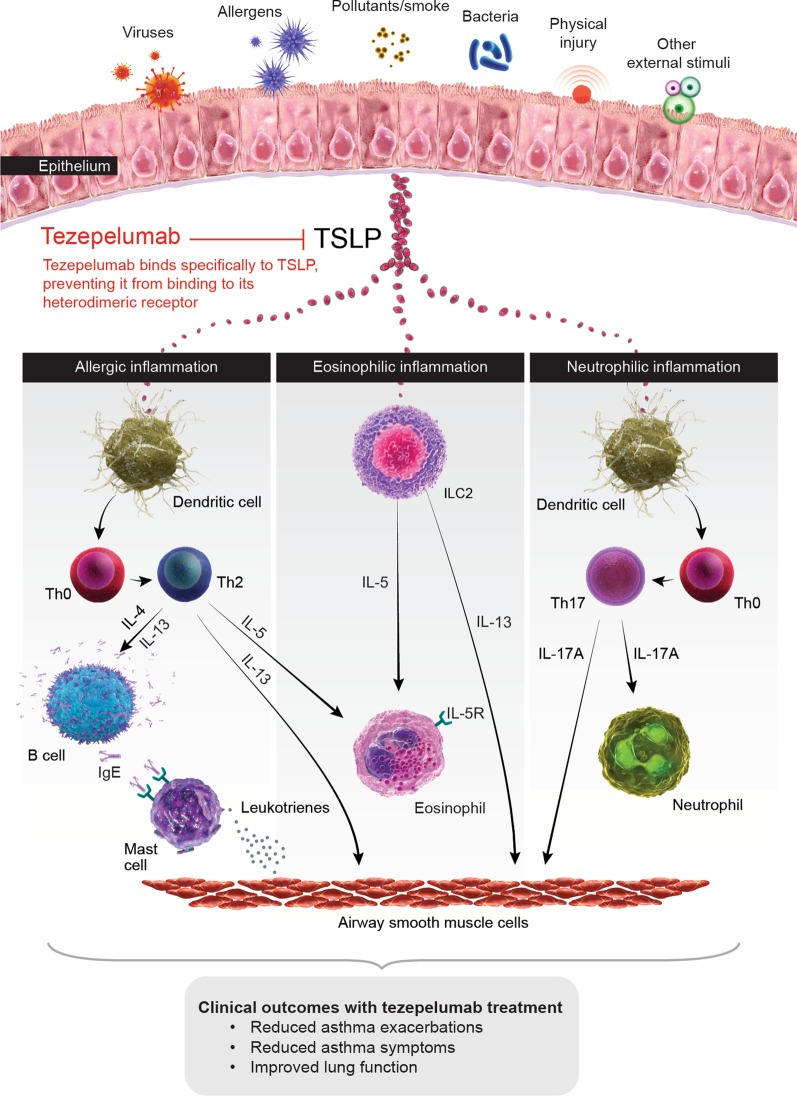
Mechanism of action by which tezepelumab improves clinical outcomes in patients with severe asthma. TSLP is released from the airway epithelium in response to insults such as viruses, allergens and pollutants, triggering multiple inflammatory cascades. Tezepelumab specifically blocks TSLP from binding to its heterodimeric receptor, thereby inhibiting the production of various inflammatory cytokines and cell types. Treatment with tezepelumab has thus far been shown to reduce blood eosinophil count, IgE, IL-5, IL-13 and FeNO. *FeNO* fractional exhaled nitric oxide, *IgE* immunoglobulin E, *IL* interleukin, *ILC2* type 2 innate lymphoid cell, *Th* T‑helper, *TSLP* thymic stromal lymphopoietin

Tezepelumab is a human monoclonal antibody (IgG2λ) that binds specifically to TSLP, blocking it from interacting with its heterodimeric receptor, thereby inhibiting the production of multiple inflammatory cytokines and activation of multiple cell types [23, 24] (Fig. 1). In the PATHWAY phase 2b study (ClinicalTrials.gov identifier: NCT02054130), treatment with tezepelumab significantly reduced asthma exacerbations by up to 71% compared with placebo in patients with severe, uncontrolled asthma, irrespective of baseline inflammation status. Improvements in lung function, asthma control and patient health-related quality of life were also observed [24, 25].

The efficacy and safety of tezepelumab are being been evaluated in an ongoing, phase 3, randomized, double-blind, placebo-controlled trial (NAVIGATOR, ClinicalTrials.gov identifier: NCT03347279), which aims to further investigate the significant reduction in asthma exacerbations observed with tezepelumab in the PATHWAY study. NAVIGATOR will also assess the effect of tezepelumab on lung function, asthma control and health-related quality of life in adults (18–80 years old) and adolescents (12–17 years old) with severe, uncontrolled asthma, irrespective of their disease phenotype. The oral corticosteroid- (OCS-) sparing potential of tezepelumab is being evaluated in an ongoing, phase 3, randomized, double-blind, placebo-controlled trial in adults with OCS-dependent asthma (SOURCE, ClinicalTrials.gov identifier: NCT03406078), for which the primary endpoint is a reduction in the prescribed daily OCS maintenance dose. Secondary objectives in the SOURCE study include assessing the effect of tezepelumab on exacerbation rate, lung function and patient-reported outcomes, while reducing the OCS dose.

Patients who complete either the NAVIGATOR or SOURCE studies are eligible to enrol in DESTINATION (ClinicalTrials.gov identifier: NCT03706079), a long-term extension (LTE) study that is evaluating the long-term safety and tolerability of tezepelumab compared with placebo, with continued dosing for approximately 1 year. During DESTINATION, study physicians have the opportunity to down- or up-titrate patients’ background medication, if appropriate. This LTE study also aims to evaluate the effect of treatment cessation after up to 2 years of tezepelumab therapy in an exploratory analysis of patients recruited from NAVIGATOR who complete tezepelumab dosing during DESTINATION. This article describes the design and objectives of the phase 3 DESTINATION study in adults and adolescents with severe, uncontrolled asthma.

## Methods

### Study design

**Fig. 2:**
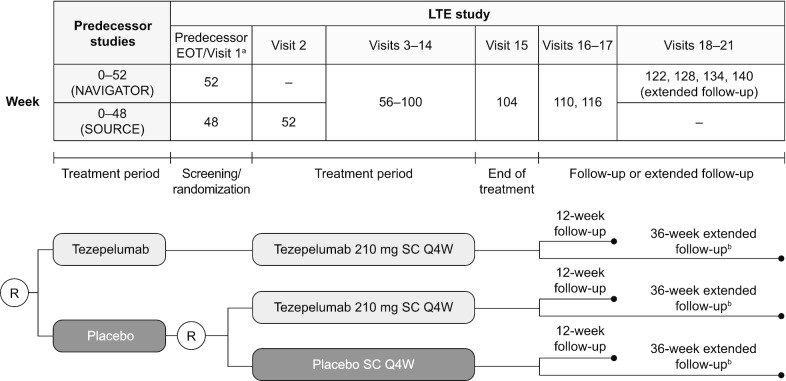
Study design. ^a^In light of the COVID-19 pandemic, the protocol was amended. Patients aiming to enrol in the DESTINATION study who are not able to attend an onsite end of treatment visit in either predecessor study will continue to participate in the 12-week safety follow-up period of either NAVIGATOR (up to week 64) or SOURCE (up to week 60) until on-site randomization and administration of the first dose of study treatment in the DESTINATION study can be conducted. ^b^Only patients from the NAVIGATOR predecessor study were eligible for a 36-week extended follow-up. *EOT* end of treatment, *LTE* long-term extension, *Q4W* every 4 weeks, *R* randomization, *SC* subcutaneously

DESTINATION is an ongoing phase 3, multicentre, randomized, double-blind, placebo-controlled, parallel-group LTE study aiming to evaluate the long-term safety and tolerability of tezepelumab 210 mg administered subcutaneously (SC) every 4 weeks (Q4W) in adults (18–80 years old) and adolescents (12–17 years old) with severe, uncontrolled asthma receiving medium- to high-dose inhaled corticosteroids (ICS) and at least one additional asthma controller with or without OCS. To be eligible for enrolment into the DESTINATION study, at screening/randomization, patients from the NAVIGATOR or SOURCE studies must have not met specific discontinuation criteria for either predecessor study and must have attended an onsite visit at the end of treatment in either predecessor study (week 52 or week 48 in NAVIGATOR or SOURCE, respectively; Fig. 2). Additional key inclusion and exclusion criteria for DESTINATION are shown in Table 1. Efficacy and safety endpoints for DESTINATION will be analysed over a 2-year period, inclusive of the treatment period of the NAVIGATOR or SOURCE predecessor studies.

**Table 1:** Key inclusion and exclusion criteria

**Key inclusion criteria at randomization**
• Female or male patients who have not met tezepelumab discontinuation criteria and have attended the end of treatment visit in either the NAVIGATOR study (NCT03347279) or the SOURCE study (NCT03406078)
**Key inclusion criteria for the extended follow-up period**
• Patients who were enrolled from the NAVIGATOR study (NCT03347279) who: – completed dosing of tezepelumab or placebo up to week 100 – did not discontinue treatment with either tezepelumab or placebo – attended the end of treatment visit (week 104)
**Key exclusion criteria at randomization**
• Pregnant, breastfeeding or lactating • Any clinically important pulmonary disease other than asthma associated with elevated peripheral eosinophil counts • Any disorder that could, in the opinion of the investigator, affect the safety of the patient throughout the study, influence the study findings or their interpretation, or impede the patient's ability to complete the study • Current malignancy or malignancy that developed during a predecessor study^a^ • Treatment with systemic immunosuppressive/immunomodulating drugs (e.g. methotrexate, cyclosporine) in the 12 weeks before randomization, with the exception of oral corticosteroids used in the treatment of asthma/asthma exacerbations • Concurrent enrolment in another clinical study involving an investigational product • Any clinically meaningful abnormal finding during the predecessor study (demonstrated by physical examination, vital signs, ECG, haematology, clinical chemistry or urinalysis) that could, in the opinion of the investigator, affect the safety of the patient throughout the study, influence the study findings or their interpretation, or impede the patient's ability to complete the study • Patients with important protocol deviations in either of the predecessor studies, assessed at the discretion of the sponsor
**Key exclusion criteria for the extended follow-up period**
• Discontinuation of tezepelumab during the treatment period of DESTINATION • Patients who were enrolled from the SOURCE study (NCT03406078)

*ECG* electrocardiogram

^a^Patients with a basal cell carcinoma or a localized squamous cell carcinoma of the skin that was resected for cure will not be excluded

DESTINATION will commence with a screening/randomization visit at the end of treatment visit of the NAVIGATOR or SOURCE predecessor studies, at which time eligible patients will receive the first LTE dose of tezepelumab or placebo. The treatment period of DESTINATION will include that of either predecessor study, and will therefore be up to 104 weeks (the last dose of study drug will be administered at week 100). Patients who were previously randomized to receive placebo Q4W in either predecessor study will be re‑randomized 1:1 at the screening/randomization visit of DESTINATION to receive either tezepelumab 210 mg Q4W SC (administered using a single-use vial and syringe) or placebo Q4W. Patients who were previously randomized to receive tezepelumab 210 mg Q4W SC in either of the predecessor studies will continue to receive tezepelumab 210 mg Q4W SC during DESTINATION. Double-blinding will be maintained using an interactive voice/web response system. Given the randomization scheme used in the predecessor studies, the resulting overall patient distribution in DESTINATION will be approximately 3:1 (tezepelumab:placebo).

Patients who complete the treatment period of DESTINATION will enter one of two follow‑up periods (during which they will stop receiving treatment). Patients recruited from NAVIGATOR will have the option to enter either a 12-week follow-up period or a 36‑week extended follow-up period. Those eligible for the extended follow-up period must complete tezepelumab or placebo dosing up to week 100, must not meet discontinuation criteria, and must attend the end of treatment visit at week 104 of DESTINATION (Table 1). Patients recruited from SOURCE will only be permitted to enter the 12-week follow-up period.

**Fig. 3:**
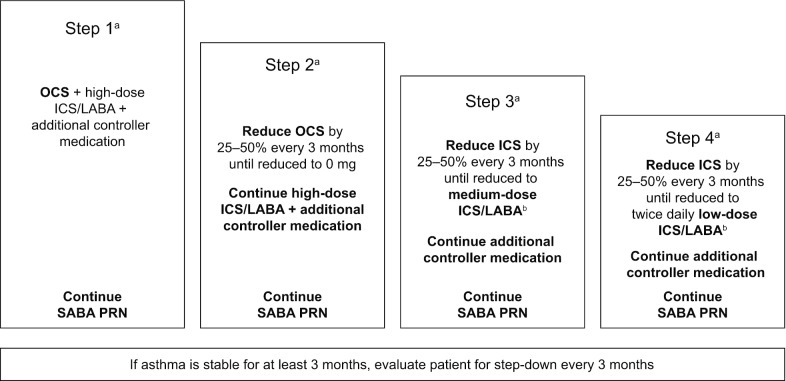
Step-down of asthma background medication. ^a^Additional controller medications can be changed or their doses can be up-titrated or down-titrated at the discretion of the investigator. ^b^If the patient is not taking a combination of ICS/LABA and is taking a separate ICS and additional controller medication(s), reduce only the dose of ICS and maintain the same dose of the additional controller medication(s). *ICS* inhaled corticosteroid, *LABA* long-acting β_2_-agonist, *OCS* oral corticosteroid, *PRN* prescribed-as-needed, *SABA* short-acting β_2_-agonist

For the duration of the study, all patients will receive their prescribed ICS plus additional controller medications (including long-acting β-agonists, leukotriene modifiers, theophylline and cromones) and investigators will be permitted to adjust these treatments as appropriate. Patients will also be permitted to use OCS for the treatment of asthma. If a patient’s asthma symptoms are well controlled and their lung function is stable for at least 3 months, starting from visit 1, investigators will have the opportunity to down- or up-titrate their background asthma medication. These modifications to patients’ background medication will be performed in accordance with Global Initiative for Asthma 2018 guidelines (Fig. 3) [26].

In light of the COVID-19 pandemic, the DESTINATION protocol has been amended to address the issue of social distancing and the possibility that site visits could be limited. Patients aiming to enrol in the DESTINATION study who are not able to attend an onsite end of treatment visit in either predecessor study will continue to participate in the 12-week safety follow-up period of either NAVIGATOR (up to week 64) or SOURCE (up to week 60) until on-site randomization and administration of the first dose of study treatment in the DESTINATION study can be conducted. The protocol has also been amended so that the first two doses of study treatment must be administered at the study site. This amendment ensures that patients who are receiving tezepelumab for the first time will undergo appropriate safety monitoring onsite. If onsite randomization and administration of the first dose of study drug in DESTINATION are not possible by the end of the safety follow-up period of either predecessor study, the patient will not be enrolled in the DESTINATION study. After the first two doses of study treatment have been administered onsite, the amendment specifically allows for telephone visits (when site visits are not possible) to collect appropriate efficacy and safety information, and for at‑home dosing of the study drug when possible.

To date (July 2020), this study has enrolled 886 patients from 182 study sites in 18 countries. Written informed consent will be obtained from all patients or their guardians before enrolment into the study and before entry into the extended follow-up period for eligible patients. The study will be conducted in accordance with the principles established in the Declaration of Helsinki and the International Council for Harmonisation guidelines for good clinical practice.

### Objectives and outcome measures

A summary of the primary and secondary objectives and endpoints for DESTINATION is given in Table 2. The primary objective is to evaluate the long-term safety and tolerability of tezepelumab over 104 weeks, inclusive of the treatment duration of the predecessor studies. Safety and tolerability will be assessed by monitoring adverse events (AEs), AEs of special interest (AESIs) and serious AEs (SAEs). The secondary objective is to evaluate the long-term effect of tezepelumab compared with placebo on asthma exacerbations, as assessed by the annualized asthma exacerbation rate (AAER), over the same 104-week period.

**Table 2:** Primary and secondary objectives and endpoints

Objective	Endpoint
*Primary objective*	
Evaluate the long-term safety and tolerability of tezepelumab in patients with severe asthma	Exposure-adjusted incidence of AEs, AESIs and SAEs over 104 weeks
*Secondary objective*	
Assess the long-term effect of tezepelumab on asthma exacerbations in adults and adolescents with severe, uncontrolled asthma, compared with placebo	AAER over 104 weeks^a^

All objectives relate to tezepelumab 210 mg Q4W SC

*AAER* annualized asthma exacerbation rate, *AE* adverse event, *AESI* adverse events of special interest, *Q4W* every 4 weeks, *SAE* serious adverse event, *SC* subcutaneously

^a^Baseline is week 0 in predecessor study (NAVIGATOR or SOURCE)

DESTINATION will investigate a number of exploratory outcomes including, but not limited to, the immunogenicity of tezepelumab at week 104 compared with baseline in the predecessor studies, as well as the effects of tezepelumab on pulmonary function, asthma control (assessed using the Asthma Control Questionnaire [ACQ]-6) and patient health status (measured using St George’s Respiratory Questionnaire). Exploratory outcomes associated with asthma exacerbations include time to first exacerbation over 104 weeks, the proportion of patients who are exacerbation-free at week 104 and the annualized rate of exacerbations associated with hospitalization, emergency department visits or urgent care visits. The effect of tezepelumab in reducing OCS dose (50% or 100% reduction) and the long-term effect of tezepelumab in maintaining OCS dose at ≤ 5 mg will be explored in patients recruited from the SOURCE predecessor study.

An additional exploratory outcome in patients who enter the 36-week extended follow-up period is the clinical effect of tezepelumab after treatment cessation. The extended follow-up period aims to evaluate whether any clinical benefits (e.g. reduction in asthma exacerbations and improvements in lung function and ACQ-6 score) and pharmacodynamic effects (e.g. changes in blood eosinophil counts, levels of fractional exhaled nitric oxide and IgE) observed with tezepelumab persist for up to 9 months after cessation of treatment. The relationship between serum tezepelumab concentrations and the effects observed after cessation of treatment will also be explored.

### Statistical considerations

No statistical hypotheses will be formally tested in this study. All data will be reported separately according to predecessor study. The safety and full analysis sets will comprise all patients who were randomized and who received at least one dose of tezepelumab or placebo in either of the predecessor studies, regardless of their enrolment into DESTINATION.

Subpopulations of patients who are enrolled in DESTINATION and who receive at least one dose of tezepelumab or placebo during this study are defined as the safety and full analysis LTE subsets. Given that the LTE subsets will comprise patients who completed the predecessor studies, they may be subject to selection bias. In particular, measures of asthma control may be affected because patients with less severe asthma may have been more likely to complete the predecessor study and enrol in the LTE study. These subpopulations will therefore be used for supportive analyses only, and the number of patients who discontinue treatment and patterns of discontinuation will be considered when interpreting the data.

The effect of tezepelumab after treatment cessation will be evaluated in an additional subpopulation comprising patients who were originally randomized in NAVIGATOR and who are in the safety analysis LTE subset, complete the LTE study and enter the extended 36-week follow-up period.

**Fig. 4:**
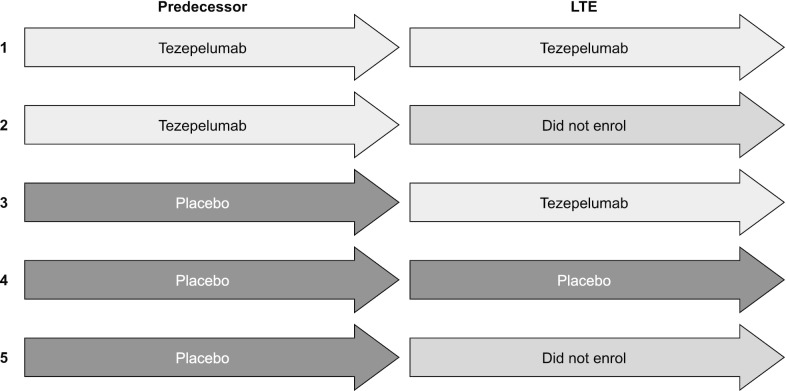
Treatment groupings across the predecessor (NAVIGATOR or SOURCE) studies and the DESTINATION LTE study. *LTE* long-term extension

There are five possible treatment groupings across the predecessor and DESTINATION studies: 1, tezepelumab-tezepelumab; 2, tezepelumab-did not enrol; 3, placebo-tezepelumab; 4, placebo-placebo; 5, placebo-did not enrol (Fig. 4). The primary treatment groups for analyses are randomized tezepelumab (all patients originally randomized to tezepelumab in the predecessor studies, i.e. groups 1 and 2) and randomized placebo (all patients originally randomized to placebo in the predecessor studies, i.e. groups 3 [until switch to tezepelumab], 4 and 5).

The supportive treatment groups are: tezepelumab predecessor plus tezepelumab LTE (all patients originally randomized to tezepelumab in the predecessor studies and re-randomized to tezepelumab in the LTE study, i.e. group 1) and placebo predecessor plus placebo LTE (all patients originally randomized to placebo in the predecessor studies and re-randomized to placebo in the LTE study, i.e. group 4) (Fig. 4). Other treatment groups will be used for exposure summaries and rare events, and to assess the durability of benefit and the effect after treatment cessation.

There will be two database locks in the DESTINATION study. The primary database lock will be conducted after the last patient completes week 104, and the final database lock will be conducted once all patients have completed the last follow-up visit at week 140. All statistical analyses of the primary and secondary efficacy outcomes will be performed based on the primary database lock data.

The baseline measurement for safety endpoints will be the last measurement on or before the first dose of study treatment in the predecessor study, and for efficacy endpoints it will be the last measurement on or before the date of randomization in the predecessor study. If there is no such value for either endpoint type, the baseline value will not be imputed, and the value will be described as ‘missing’. Statistical analyses of safety outcomes will be performed on the safety analysis set, and key safety analyses will be repeated using the safety analysis LTE subset.

AEs, AESIs and SAEs, which will be used to assess the primary objective, will be summarized for the safety analysis set over the 104-week study period using exposure-adjusted incidence (i.e. number of patients reporting events divided by person–time at risk) to account for the variability in follow-up. In addition, exposure-adjusted incidence will be explored over defined time periods to assess potential changes in risks with longer exposure to treatments. Observed and change-from-baseline values in laboratory data will be summarized descriptively.

Statistical analyses of efficacy outcomes will be performed primarily on the full analysis set; key efficacy analyses may be repeated using the full analysis LTE subset. The secondary objective will be assessed by comparing the AAER over 104 weeks in the tezepelumab group with that of the placebo group. This will be achieved by using a negative binomial model with the total number of asthma exacerbations experienced from baseline in the predecessor studies until week 104 in the LTE study as a response variable. Treatment, history of exacerbations and stratifying variables from the predecessor studies will be included as covariates in the model. The logarithm of the time at risk for exacerbation during the study will be used as an offset variable in the model.

Exploratory outcomes will be summarized descriptively; for the effect of tezepelumab after cessation of treatment, efficacy data will be presented using descriptive statistics by time period, and safety data will be summarized by time period using exposure-adjusted incidence to account for the variability in follow-up. Laboratory data will be summarized descriptively over time. Loss of clinical effect will be explored through assessments including initiation of another biologic treatment, increase in the OCS maintenance dose, increase in symptoms measured by the ACQ, asthma exacerbations and study withdrawal. Any additional statistical analyses required owing to COVID-19 will be pre-specified in the statistical analyses plan before sponsor unblinding and will be described when the results for this study are reported.

## Discussion

DESTINATION aims to evaluate the long-term safety, tolerability and efficacy of tezepelumab compared with placebo in patients with severe, uncontrolled asthma who complete the NAVIGATOR or SOURCE predecessor studies and continue dosing for approximately 1 year. The effect of treatment cessation after up to 2 years of tezepelumab therapy will also be assessed in patients who enrol from NAVIGATOR and complete treatment dosing during DESTINATION.

In DESTINATION, patients who are randomized to receive placebo in one of the predecessor studies will be re-randomized to receive either tezepelumab or placebo, resulting in an overall patient distribution of approximately 3:1 (tezepelumab:placebo). The placebo-controlled study design of DESTINATION will help to contextualize the endpoints by providing a direct comparator to the data collected from patients randomized to receive tezepelumab.

A novel aspect of DESTINATION is the exploratory evaluation of the effect of tezepelumab after treatment cessation during a 36-week extended follow-up period. The duration of this period is based on findings from pharmacokinetic and pharmacodynamic modelling, which indicate that tezepelumab serum concentrations are expected to decrease below those required for a minimal pharmacodynamic effect (< 10% of the maximum effect) 20–27 weeks after cessation of treatment for fractional exhaled nitric oxide and 27–33 weeks after cessation of treatment for blood eosinophil counts. The extended follow-up period aims to characterize the clinical changes and changes in asthma biomarkers that occur off-treatment, beyond the expected pharmacokinetic and pharmacodynamic effects. Potential correlations between clinical signs and symptoms of asthma and changes in biomarkers will help to further our understanding of the mechanism of action of tezepelumab and the duration of its effects after treatment cessation. There are limited data from clinical studies on the effect of available biologics after treatment cessation; outcomes following mepolizumab discontinuation have been evaluated in a post hoc analysis of an open-label, phase 3b trial [27] and in a 12‑month unblinded, prospective, observational extension study [28].

In the DESTINATION study, investigators have the opportunity to down-titrate their patients’ background asthma medication (in accordance with Global Initiative for Asthma 2018 guidelines [26]) if their asthma symptoms are well controlled and their lung function is stable for at least 3 months. Investigators may also up-titrate background medication if their patients’ asthma symptoms are not well controlled and their lung function is unstable. It is hypothesized that continued treatment with tezepelumab may allow patients to reduce their use of prescribed controller therapies or rescue medication. This approach has also been taken in clinical studies with other biologics in patients with severe asthma [3, 10, 29, 30].

The primary objective of DESTINATION is to evaluate the long-term safety and tolerability of tezepelumab; the dosage (210 mg Q4W SC) is the same as that given in the predecessor studies. Per standard practice, safety and tolerability will be assessed by monitoring AEs, AESIs and SAEs. The secondary endpoint will assess the effect of tezepelumab on asthma exacerbations using the AAER, which is a well-accepted efficacy measure for studies of patients with severe asthma. Indeed, the benefits of tezepelumab compared with placebo were clearly demonstrated using this endpoint in the phase 2b PATHWAY study [24]. DESTINATION aims to provide valuable information about the long-term safety of tezepelumab, its effect after cessation of treatment and its clinical effect on exacerbations in patients with severe, uncontrolled asthma.

## Conclusions

DESTINATION is an LTE study that is evaluating the long-term safety and tolerability of tezepelumab versus placebo, with continued dosing for approximately 1 year after patients with severe, uncontrolled asthma complete the phase 3 NAVIGATOR or SOURCE tezepelumab studies. In DESTINATION, investigators have the opportunity to down- or up-titrate patients’ background medication during treatment with tezepelumab. In addition, an exploratory analysis will assess the effect of tezepelumab on asthma exacerbations, lung function and inflammatory biomarkers after cessation of treatment, and will provide important new insights into the long-term safety and efficacy of this biologic. DESTINATION builds on previous clinical findings with tezepelumab and aims to support its potential long-term treatment benefits in patients with severe, uncontrolled asthma.

## Data Availability

The datasets used and/or analysed during the current study are available from the corresponding author on reasonable request.

## Abbreviations

AAER: Annualized asthma exacerbation rate
ACQ: Asthma Control Questionnaire
AE: Adverse event
AESI: Adverse event of special interest
ICS: Inhaled corticosteroid
Ig: Immunoglobulin
IL: Interleukin
LTE: Long-term extension
OCS: Oral corticosteroid
Q4W: Every 4 weeks
SAE: Serious adverse event
T2: Type 2
TSLP: Thymic stromal lymphopoietin
